# Attention-Deficit/Hyperactivity Disorder and Unhealthy Lifestyle in Adolescence: Unforeseen Role of Allostatic Overload and Psychological Well-Being

**DOI:** 10.3390/healthcare12100956

**Published:** 2024-05-07

**Authors:** Sara Gostoli, Giulia Raimondi, Chiara Rafanelli, Paola Gremigni

**Affiliations:** Department of Psychology “Renzo Canestrari”, University of Bologna, 40127 Bologna, Italy; giuliaraimondi.dr@gmail.com (G.R.); chiara.rafanelli@unibo.it (C.R.); paola.gremigni2@unibo.it (P.G.)

**Keywords:** ADHD, adolescence, allostatic overload, moderation analysis, psychological well-being, unhealthy behaviors

## Abstract

Unhealthy lifestyle behaviors (ULBs) are common in early adolescence and could be worsened by Attention-Deficit/Hyperactivity Disorder (ADHD), as well as by specific psychosocial factors, such as stress and unbalanced (i.e., too high or low scores of) psychological well-being (PWB) dimensions. This multi-center study aimed to evaluate how interactions between ADHD symptoms and psychosocial factors associated with ULBs (i.e., Allostatic Overload and multidimensional Psychological Well-Being), considered as moderators, could affect the adoption of ULBs during adolescence. A total of 440 fourteen-year-old adolescents were recruited from six upper secondary schools in Bologna and Rome (Italy) and completed self-report questionnaires on ULBs, ADHD, and psychosocial factors. Relations between ADHD symptomatology and specific ULBs (i.e., impaired sleep, problematic Internet use) were moderated by variables deemed as “negative” (i.e., Allostatic Overload) or “positive” (i.e., PWB dimensions of Self-Acceptance, Personal Growth, Positive Relations, Purpose in Life, Environmental Mastery): when the “negative” moderator is absent and the levels of the “positive” moderators are higher, ULBs decrease among students with lower ADHD symptomatology but increase among students with more severe ADHD. Based on ADHD severity, interventions should aim at promoting a state of euthymia, which consists in balanced PWB dimensions and reflects the optimal level of well-being to fulfill one’s own potential and self-realization.

## 1. Introduction

Adolescence is a developmental phase characterized by major biological and psychosocial changes; thus, it is crucial to preserve or promote a good health status to people in this stage in order to prevent consequent impairments. Unhealthy lifestyle behaviors (ULBs) identify a broad category of detrimental everyday habits such as, for example, smoking, lack of sleep, or Internet use, which may affect both physical and mental health [1]. In the present study, ULBs specifically include substance use, poor quality of sleep, and problematic utilization of technological devices. The adoption of ULBs is frequent during adolescence, mostly due to social pressure among peers [2]. For instance, sleep problems and technology addiction are quite common among adolescents [3]. Furthermore, the adoption of ULBs during adolescence constitutes an important risk factor for the development of psychiatric disorders [4] and non-communicable diseases (i.e., medical illnesses typically caused by modifiable environmental etiological factors) in adulthood [5].

Psychosocial factors associated with the adoption of an unhealthy lifestyle in adolescence have been investigated. For example, it has been proven that stress could represent a risk factor since it is associated with ULBs, such as those leading to obesity, among children and adolescents [6] and plays a moderating role on different ULBs (e.g., smoke, alcohol use, poor sleep, problematic Internet use) in adolescence and adulthood [7,8,9,10]. Moreover, when the continuous or repeated exposure to stress exceeds the individual’s coping strategies, it might ensue allostatic overload. This psychosomatic syndrome represents the “cost” of chronic exposure to fluctuating or heightened neural or neuroendocrine responses [11] and reflects the cumulative effects of experiences in daily life involving both ordinary events and major challenges, including the physiological consequences of ULBs [12]. When either uncontrolled/undesirable or subtle/long-standing stressors are experienced as taxing or exceeding own coping skills and, at the same time, the individual develops consequent psychological symptoms, social and/or psychological well-being impairments, allostatic overload can be diagnosed. It has been associated with severe health diseases during adulthood [12] but has been scarcely investigated among adolescents. Another factor that has been associated with the adoption of ULBs is psychological well-being (PWB) that, according to Ryff’s model [13], encompasses six dimensions: self-acceptance, positive relationships, purpose in life, environmental mastery, personal growth, and autonomy. Although PWB is often regarded as a protective factor, some studies have shown that unbalanced levels of PWB, namely either too high or low scores in one or more of its six dimensions, are associated with ULBs, both in adolescents and adults [14,15], and that PWB seems to play a moderating role in problematic Internet gaming [16] in adulthood. 

Attention-Deficit/Hyperactivity Disorder (ADHD) is a neurodevelopmental disorder characterized by impaired attention (i.e., reduced ability to keep focus), hyperactivity (i.e., excessive movement, inadequate to the setting), or impulsivity (i.e., acting on impulse without thinking) that does not align with the individual’s developmental stage and negatively affects social and academic functioning [17]. It often lasts into adulthood and has been associated with severe psychopathological outcomes [18,19]. Usually, the clinical diagnosis of ADHD is based on a clinical interview with the patient, as well as on information request from other significant people (e.g., family, teachers), specifically conducted by a trained psychiatrist and/or clinical psychologist [20]. However, in research settings, self-report instruments on ADHD symptomatology are often the measures of choice due to their easy administrability, especially in large-scale studies. Several investigations have shown that ULBs are worsened in adolescents with ADHD [21,22]. In particular, smartphone addiction and sleep problems were more severe in adolescents with ADHD compared to individuals without ADHD [22,23].

While previous research has extensively examined the specific contributions of ADHD symptoms and psychosocial factors (e.g., stress-related syndrome and psychological well-being) to the adoption of ULBs, there is still a remarkable gap in the literature on how these factors interact and potentially exacerbate or mitigate the likelihood of engaging in unhealthy behaviors. This could help to better identify potential risk or protective factors for ULBs and give indications for the development of effective prevention programs in school settings.

The present study aimed to evaluate how the interactions between ADHD symptoms and psychosocial factors could affect the adoption of ULBs (i.e., substance use, poor quality of sleep, and problematic technological devices utilization) during adolescence in a non-clinical sample of first-year students at upper secondary schools. Specifically, the objective of the present investigation was to evaluate whether the presence of risk and protective factors, namely allostatic overload and impaired/balanced psychological well-being dimensions, could moderate the relationship between ADHD symptomatology and ULBs.

## 2. Materials and Methods

### 2.1. Design and Procedures

**Figure A1:**
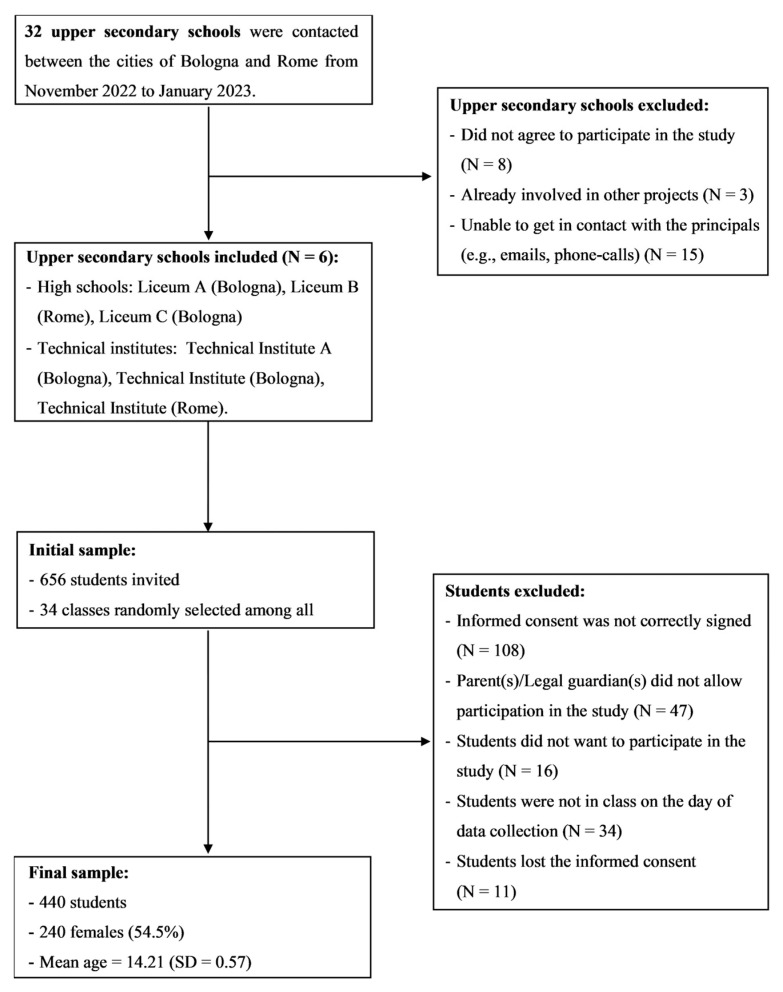
Flow chart of study phases.

For the purposes of the present multi-center cross-sectional study, a total of 32 upper secondary schools were contacted in Bologna and Rome (Italy) from November 2022 to January 2023. Six schools agreed to participate and 34 (out of 71) first-year classes were chosen through a random number generator. The entire enrollment process is described in Figure A1 (Appendix A).

During the enrollment period (January–April 2023), each student was given an informed consent form, which required the signature of both parents or legal guardian(s) to participate in the study. Then, students were given one week to return the signed consent forms before data collection began. All participants were made aware that their participation was voluntary and anonymous, and that they would not receive any form of compensation (i.e., school credits) for their participation in the study. Students whose consent forms were not correctly signed were unable to take part in the study and were given supervised school assignments. 

The questionnaires were made available to the students through an online platform called Microsoft Forms. Each student included in the study was provided with a QR code, which they had to scan using their smartphone to access the link to the questionnaires. In cases where Internet services and/or a smartphone were not available, the students were given a printed version of the questionnaires to complete. The entire process, including accessing the questionnaires and completing them, took approximately one hour.

The collected data have been downloaded into a personal computer, protecting the dataset with a keyword known only to the research group. According to the Italian deontological code, the mentioned data will be stored for up to five years from collection. The identity of participants, associate names, contacts, or personal information were not trackable in any way.

### 2.2. Sample

Participants were admitted in the present study if they: (a) were students attending the first year of the selected schools; (b) had informed consent signed by both parents/legal guardian(s); (c) gave their informed assent. A total of 440 adolescents participated in the study.

### 2.3. Assessment Instruments

#### 2.3.1. ULBs

Substances use. The Italian version [24] of the Alcohol Use Disorder Identification Test-Consumption (AUDIT-C) [25] was used to assess problematic alcohol use. AUDIT-C includes 3 items (i.e., “*How often did you have a drink containing alcohol in the past year?*”; “*How many drinks did you have on a typical day when you were drinking in the past year?*”; “*How often did you have six or more drinks on one occasion in the past year?*”) rated on a 5-point response level of increasing severity. Total score may range from 0 to 12, with higher scores indicating more severe alcohol use. In the current study, the total score yielded an acceptable Cronbach’s alpha of 0.68 [26].

The Italian translation of the Heaviness of Smoking Index (HIS) [27] was used to assess problematic smoking behavior. HSI is composed of two items (i.e., “*How soon after you wake up do you smoke your first cigarette?*”, “*How many cigarettes/day do you smoke?*”). Total score may range from 0 to 9, with higher scores indicating more severe smoking. In the current study, the total score yielded an acceptable Cronbach’s alpha of 0.67 [26].

The Italian validated version [28] of the Cannabis Abuse Screening Test (CAST) [29] was used to assess problematic patterns of cannabis use. The first part of the questionnaire evaluates cannabis use in the whole life, in the past 12 months and/or in the past 30 days. Additional six questions on problems related to cannabis use (e.g., “*Have you had memory problems when you smoked cannabis?*”; “*Have you tried to reduce or stop your cannabis use without succeeding?*”) are asked. These six items are rated on a 5-point Likert scale. The Italian version [28] of the total CAST score may range from 0 to 19, with higher scores indicating more problematic cannabis use. It demonstrated satisfactory discriminant validity (i.e., ROC curve) in categorizing individuals with and without cannabis dependence [28]. In the current study, the total score yielded a good Cronbach’s alpha of 0.77 [26].

Sleep. A single item assessing mean hours of sleep each night (i.e., “*On average, how many hours do you sleep each night?*”) and another investigating the amount of time spent using technological devices instead of sleeping (i.e., “*Thinking about the technological device you use the most, how many hours do you spend using it every night instead of sleeping?*”), were included. An item derived from the Pittsburgh Sleep Quality Index [30], rated on a 4-point Likert scale, was used to assess quality of sleep during the past month. In this case, a higher score corresponds to worse quality of sleep. 

Technological devices use. The Italian version [31] of the Internet Addiction Test (IAT) [32] was used to assess Internet use. IAT includes twenty items rated on a 5-point Likert scale and encompasses six factors: (1) Compromised Social Quality of Life-CSQL (i.e., social life impairment due to Internet use; e.g., “*How often do others in your life complain to you about the amount of time you spend on-line?*”); (2) Compromised Individual Quality of Life-CIQL (i.e., individuals’ activities impairment due to Internet use; e.g., “*How often do you neglect household chores to spend more time on-line?*”); (3) Compensatory Usage of the Internet-CUI (i.e., anticipatory need of Internet use; e.g., “*How often do you find yourself anticipating when you will go on-line again?*”); (4) Compromised Academic/Working Careers-CAWC (i.e., academic/work careers impairment due to Internet use; e.g., “*How often do your grades or schoolwork suffer because of the amount of time you spend on-line?*”); (5) Compromised Time Control-CTC (i.e., incapacity to stop Internet use; e.g., “*How often do you find that you stay on-line longer than you intended?*”); (6) Excitatory Usage of the Internet-EUI (i.e., excitement associated with going online; e.g., “*How often do you block out disturbing thoughts about your life with soothing thoughts of the Internet?*”). Higher scores in each dimension indicate greater Internet use. A global score for Internet addiction can be obtained by averaging and summing the scores obtained at each subscale. The Italian version of the IAT was found to have good psychometric properties [33]. In the current study, the total score yielded an excellent Cronbach’s alpha of 0.89 [26].

#### 2.3.2. ADHD

The Italian version [34] of the Adult ADHD Self-Report Scale (ASRS) [35] was used to assess the levels of ADHD-related symptoms in terms of frequency. ASRS includes eighteen items (e.g., “*How often do you have trouble wrapping up the final details of a project, once the challenging parts have been done?*”; “*How often do you have difficulty getting things in order when you have to do a task that requires organization?*”; “*How often do you have problems remembering appointments or obligations?*”; “*When you have a task that requires a lot of thought, how often do you avoid or delay getting started?*”; “*How often do you fidget or squirm with your hands or feet when you have to sit down for a long time?*”; “*How often do you feel overly active and compelled to do things, like you were driven by a motor?*”) rated on a 5-point Likert scale and allows a total ADHD score, which may range from 0 to 72; higher scores indicating greater frequency and thus the severity of ADHD symptoms. ASRS has already been used with adolescents [34,36] and has demonstrated good psychometric properties (i.e., internal consistency, sensitivity, and specificity) [36]. In the current study, the total score yielded an excellent Cronbach’s alpha of 0.84 [26].

#### 2.3.3. Psychosocial Factors

Allostatic overload. PsychoSocial Index-Young (PSI-Y) [37] was used to assess the presence of allostatic overload. The PSI-Y is derived from the original version of the PsychoSocial Index [38] and it is specifically used to assess psychosocial factors among adolescents and young adults. The PSI-Y includes 40 items, with 21 items scoring on a dichotomous format (Yes/No), 18 items on a 4-point Likert scale (from 1 “not at all” to 4 “a great deal”), and a final item assessing Quality of Life scoring on a 5-point Likert scale (from 0 “Excellent” to 4 “Awful”). It encompasses different domains: Stress (scores ranging from 0 to 15), Psychological Distress (scores ranging from 0 to 45), Abnormal Illness Behavior (scores ranging from 0 to 9), Psychological Well-Being (scores ranging from 0 to 6), and Quality of Life (scores ranging from 0 to 4). For the determination of Allostatic Overload, only stress, psychological distress, and psychological well-being domains have been used [37]. The operationalization of Allostatic Overload is based on specific clinimetric criteria [12], which require the presence of an identifiable stressor that must be judged as exceeding/taxing the individual’s coping skills (Criterion A) and psychiatric and/or psychosomatic symptoms, impaired functioning, and/or compromised well-being (Criterion B).

Psychological well-being. The 18-item Italian version [39] of the Psychological Well-Being scales (PWBs) [13] was used to assess psychological well-being according to Ryff’s multidimensional model, including: Self-acceptance (e.g., “*I like most aspects of my personality*”), Positive Relationships (e.g., “*I know I can trust my friends, and they know they can trust me*”), Purpose in Life (e.g., “*I am an active person in carrying out the plans I set for myself*”), Environmental Mastery (e.g., “*I am good at juggling my time so that I can fit everything in that needs to get done*”), Personal Growth (e.g., “*In general, I feel that I continue to learn more about myself as time goes by*”), and Autonomy (e.g., “*I am not afraid to voice my opinions, even when they are in opposition to the opinions of most people*”). Items are rated on a 4-point Likert scale and higher scores indicate greater psychological well-being. Internal consistency among Italian youth population has demonstrated to be satisfactory [40,41]. In the current study, the total score yielded an acceptable Cronbach’s alpha of 0.62 [26].

### 2.4. Statistical Analysis

Means (SD) or percentage for continuous and categorical variables, respectively, were calculated as descriptive statistics. General Linear Models (GLM) were used to assess the possible moderating roles of allostatic overload and psychological well-being in the relationship between ADHD symptomatology and ULBs. 

The *p* value was set at <0.05. All the analyses were performed with the package General Analyses for Linear Model (GALMj) [42] for Jamovi (version 2.3) [43]. 

## 3. Results

The present investigation revealed that allostatic overload and specific psychological well-being dimensions play a significant moderating role in the relationship between ADHD symptomatology and ULBs. Detailed descriptive statistics of the sample are reported in Table 1.

### 3.1. Substances Use

Concerning alcohol use, a main effect of ADHD symptoms was found (F_(1,439)_ = 7.08, *p* < 0.01), with a greater ASRS score predicting greater alcohol use (β = 0.18, *p* < 0.01). However, no significant interaction effect was detected between ASRS score and any of the psychosocial factors considered as potential moderators. 

ASRS did not yield any significant main effect on cigarette (F_(1,439)_ = 1.62, *p* = 0.20) and cannabis use (F_(1,439)_ = 1.48, *p* = 0.23), and no significant interaction effects were detected with the considered psychosocial factors.

### 3.2. Sleep

**Figure 1:**
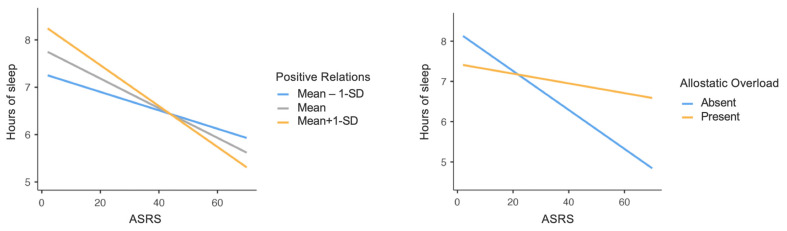
Graphs representing ASRS total score × PWB Positive Relations and ASRS total score × Allostatic Overload interactions, concerning hours of sleep. Note: ASRS = Adult ADHD Self-Report Scale; PWB = Psychological Well-Being scales; SD = Standard Deviation.

Significant interaction effects of Allostatic Overload (F_(1,431)_ = 11.58, *p* < 0.01) and PWB Positive Relations dimension (F_(1,431)_ = 4.74, *p* = 0.03) were found in the relationship between ASRS total score and mean hours of sleep. Specifically, at the absence of Allostatic Overload and higher levels of PWB Positive Relations, the effect of ADHD symptomatology on mean hours of sleep was stronger; in other words, higher ADHD symptoms severity reduced hours of sleep, and this reduction was stronger when Allostatic Overload was absent and the levels of PWB Positive Relations were higher (Figure 1).

Concerning quality of sleep, a main effect of ADHD symptoms was found (F_(1,439)_ = 5.29, *p* = 0.02), with lower ASRS scores predicting better quality of sleep (β = −0.11, *p* = 0.02). However, no significant interaction effect was detected between ASRS score and any of the psychosocial factors considered. 

**Figure 2:**
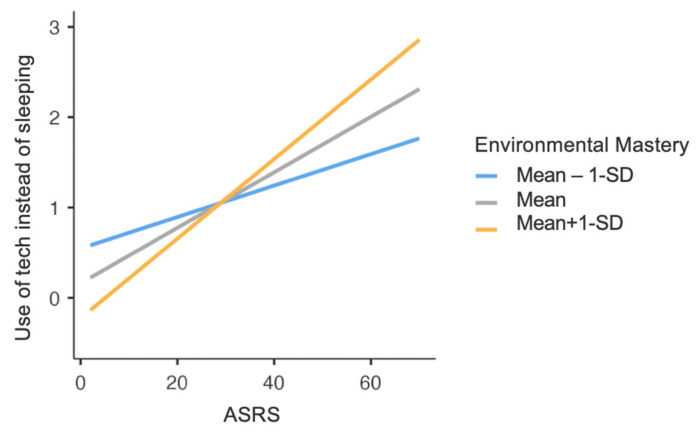
Graph representing ASRS total score × PWB Environmental Mastery interaction, concerning daily hours spent using technological devices instead of sleeping. Note: ASRS = Adult ADHD Self-Report Scale; PWB = Psychological Well-Being scales; SD = Standard Deviation.

Finally, a significant moderation effect (F_(1,432)_ = 5.28, *p* = 0.02) of PWB Environmental Mastery dimension was found in the relationship between ASRS total score and mean hours spent using technological devices instead of sleeping. Specifically, at higher levels of PWB Environmental Mastery, the effect of ADHD symptomatology on the mean hours spent using technological devices instead of sleeping was stronger; in other words, higher ADHD symptoms severity increased the time spent using technological devices instead of sleeping, and this increase was stronger when the levels of PWB Environmental Mastery were higher (Figure 2).

### 3.3. Technological Devices Use

**Figure 3:**
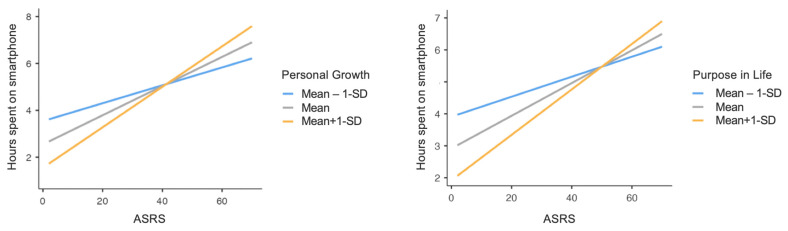
Graphs representing ASRS total score × PWB Personal Growth and ASRS total score×PWB Purpose in Life interactions, concerning daily hours spent using smartphone. Note: ASRS = Adult ADHD Self-Report Scale; PWB = Psychological Well-Being scales; SD = Standard Deviation.

Significant PWB Personal Growth and Purpose in Life dimensions significantly moderated the relationship between ASRS total score and hours spent using smartphones. Indeed, the interaction between PWB Personal Growth and ASRS total score was statistically significant (F_(1,431)_ = 5.56, *p* = 0.01) as well as that between PWB Purpose in Life and ASRS total score (F_(1,431)_ = 4.04, *p* = 0.04). Specifically, at higher levels of PWB Personal Growth and Purpose in Life, the effect of ADHD symptomatology on hours spent using smartphones was stronger; in other words, higher ADHD symptoms severity increased the time spent using smartphones, and this increase was stronger when the levels of PWB Personal Growth and Purpose in Life were higher (Figure 3).

**Figure 4:**
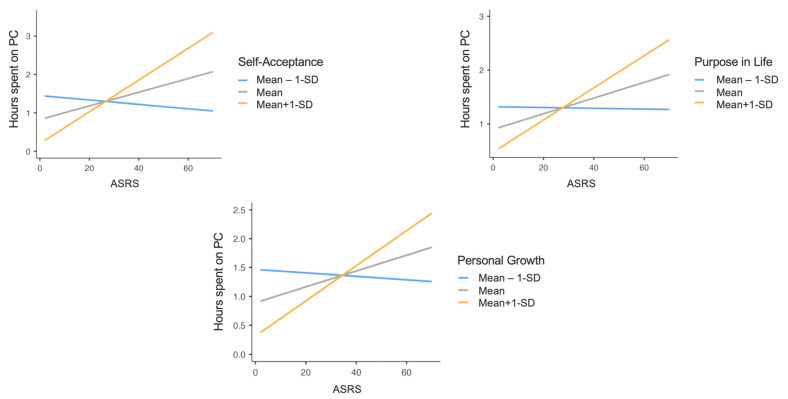
Graphs representing ASRS total score × PWB Self-Acceptance, ASRS total score × PWB Personal Growth and ASRS total score × PWB Purpose in Life interactions, concerning daily hours spent using personal computers. Note: ASRS = Adult ADHD Self-Report Scale; PWB = Psychological Well-Being scales; SD = Standard Deviation.

Furthermore, significant interaction effects of PWB dimensions of Self-acceptance (F_(1,433)_ = 10.64, *p* = 0.01), Personal Growth (F_(1,433)_ = 5.59, *p* = 0.01), and Purpose in Life (F_(1,433)_ = 4.86, *p* = 0.02) were found in the relationship between ASRS total score and hours spent using PCs. Specifically, at higher levels of PWB Self-acceptance, Personal Growth, and Purpose in Life, the positive effect of ADHD symptomatology on time spent using PCs was stronger; in other words, higher ADHD symptoms severity increased time spent on PCs, and this increase was stronger when the levels of PWB dimensions were higher (Figure 4).

**Figure 5:**
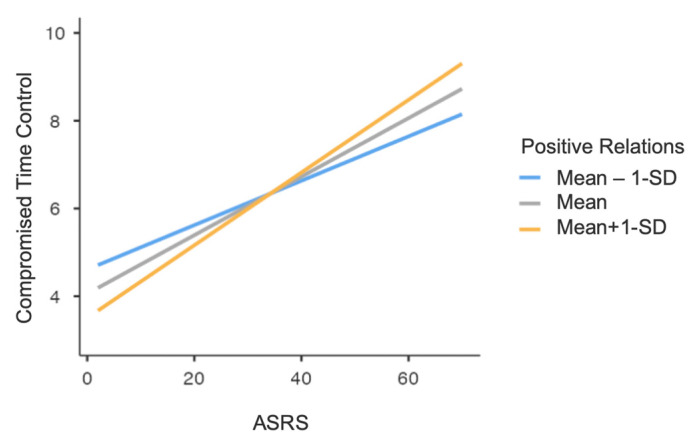
Graph representing ASRS total score × PWB Positive Relations interaction, concerning IAT Compromised Time Control (CTC). Note: ASRS = Adult ADHD Self-Report Scale; IAT = Internet Addiction Test; PWB = Psychological Well-Being scales; SD = Standard Deviation.

Finally, a significant moderation effect of PWB Positive Relations was found in the association between ADHD symptomatology and the CTC dimension of the IAT (F_(1,439)_ = 4.14, *p* = 0.04). Specifically, at a higher level of PWB Positive Relations, the effect of ADHD symptoms on time control related to Internet use was higher; in other words, higher ADHD symptoms severity increased IAT Compromised Time Control level, and this effect was stronger when PWB Positive Relations scores were higher (Figure 5). 

Correlations among variables are reported in Appendix A (see Table A1).

## 4. Discussion

The aim of the current study was to assess the role of specific psychosocial factors in moderating the relations between ADHD symptomatology and certain ULBs that are common among adolescents attending the first year of upper secondary school. 

Our results indicated that ADHD symptomatology did not significantly predict cigarette and cannabis use, differently from the literature [44]. A possible explanation could be supported by the findings of Rhodes et al. [45]. The authors found that adolescents with ADHD were prone to become regular smokers more quickly than their peers without ADHD, whereas no differences in terms of number of cigarettes smoked per day have been found. In other words, ADHD seems to be crucial in the chronicization of this unhealthy behavior, but—once individuals become regular smokers—the ADHD component does not seem to affect the extent of smoking. Since the fact of being a regular smoker was not assessed in the present study, this could explain why our analyses did not yield significant results. 

On the contrary, ADHD symptoms were significant predictors of alcohol use and quality of sleep, in line with literature [22], although without any moderation effect by the psychosocial factors considered (i.e., Allostatic Overload and Psychological Well-Being dimensions). 

The relation between ADHD symptomatology and other ULBs, such as mean hours of sleep, time spent using technological devices instead of sleeping, and problematic Internet use, was moderated by six different variables among PSI-Y domains (i.e., Allostatic Overload) and PWB dimensions (i.e., Self-acceptance, Personal Growth, Positive Relations, Purpose in Life, Environmental Mastery).

As expected, when ADHD symptomatology is lower or absent, both the absence of a moderator usually considered as “negative” or a risk factor (i.e., Allostatic Overload) and higher levels of moderators considered as “positive” or protective factors (i.e., Self-acceptance, Personal Growth, Positive Relations, Purpose in Life, Environmental Mastery) reduced the negative effect of ADHD symptomatology on ULBs by acting as buffers. These results are in line with previous studies on adolescents without ADHD, indicating that both individual and psychosocial factors (i.e., social problems, somatic behaviors) are associated with unhealthy lifestyle [1].

However, the results were reversed when ADHD symptomatology increases. Indeed, both the absence of allostatic overload and higher levels of psychological well-being dimensions enhanced the negative effect of ADHD symptomatology on the adoption of ULBs, thus representing risky instead of protective factors. Even though these results might seem to be in contrast with expectations, they are in line with a body of research showing that higher levels of psychological well-being are associated with worst outcomes in terms of ULBs, such as lack of weight loss in obese patients [15] and binge drinking [14]. Gostoli et al. [14] found that higher PWB Positive Relations levels were associated with a more problematic alcohol use among young adolescents. Although in the mentioned study ADHD symptomatology was not investigated, its presence and influence could be hypothesized in the light of the findings of the present study, indicating that having elevated levels of psychological well-being could be as disruptive as having low levels when ADHD severity increases. 

A possible explanation of how and why high levels of psychological well-being can interact in such a way with ADHD comes from neuro-connectivity research. Studies reported a strong association between ADHD and the behavioral activation system (BAS) (i.e., related to reward activity and behaviors and physiological arousal) [46], which has been associated with greater left frontal alpha asymmetry. The latter has been associated with higher levels of psychological well-being [47]. This means that clinical ADHD and too high psychological well-being levels might share a common neural activation, which could explain why our results indicated that for higher ADHD symptomatology, higher levels of psychological well-being are linked to more frequent ULBs, since such ULBs (e.g., use of technological devices) are rewarding behaviors.

Our results also suggested that reduced hours of sleep are associated with the absence of allostatic overload in adolescents with higher levels of ADHD symptoms. Allostatic overload is a psychosomatic condition that is caused by the presence of chronic or repeated stress. Therefore, since previous studies reported a hypoactive hypothalamus-pituitary-adrenal (HPA) axis with associated lower levels of cortisol in children with ADHD, also known as the “stress hormone” [48], it can be hypothesized that low levels of cortisol could reflect a reduced ability to respond to environmental demands [49], which thus could explain the present findings.

In our study, PWB Positive Relations moderated the relations of ADHD with both hours of sleep and the compromised time control aspect of Internet use. A recent study [50] on adolescents found no difference between young adolescents with and without ADHD in terms of quality of friendship and negative interactions with peers. It has also been demonstrated that children with ADHD tend to have more interest in friendships, mainly for entertainment and amusement, in comparison with their peers without ADHD, who instead crave caregiving and intimacy [51]. Lastly, even though the Internet (i.e., social media) can be used as a tool to have fun with friends, it has also been proven that using Internet to communicate with peers can foster Internet addiction [52], which in turn can affect the amount of sleep [53]. Such findings could explain why our results suggest that at high levels of positive relations, there was an increase in compromised time control related to Internet use and a reduction of hours of sleep associated with higher levels of ADHD symptoms. In other words, having online positive relations with peers could lead to more time spent online and, consequently, diminishing time control and amount of sleep. In addition, studies proved that problematic phone use is associated with ADHD symptoms as well [54]. A possible explanation is based on the need of new sensation-seeking, which refers to the search for novel and complex experiences/sensations and willingness to take risks to achieve a sense of vitality that is typical of the persons with ADHD [55]. This definition partially overlaps the characteristics of individuals with too high levels of personal growth (i.e., being unable to process negativity and inclined to set unrealistic standards for overcoming adversities) and purpose in life (i.e., having obsessional passions, being unable to admit failures and to change perspectives and goals) [13,56]. Therefore, having high sensation seeking could be associated with one’s own feeling of personal growth and purpose in life, which could explain why high levels of these two dimensions strengthened the detrimental effect of ADHD on smartphone and PC use. The search for new complex experiences and sensation is also related to having too high environmental mastery (i.e., having the tendency to get into difficult situations, being unable to savor positive emotions and relax) [13,56]. This seems to be referred also to multitasking behaviors in ADHD [17,57]. Therefore, being able to multitask could be connected to having a high sense of environmental mastery, which in turn makes it difficult for individuals with ADHD to be able to stop using technological devices and go to sleep, as found from our results.

A qualitative study [49] showed that adolescents with ADHD seem to practice acceptance to reduce the stress derived by environmental demands. This could indicate that adolescents with higher ADHD symptoms might be somewhat aware of their difficulties (i.e., need to spend too many hours using technological devices) and limitations and just accept them instead of trying to reduce their unhealthy behaviors. Therefore, this could explain why our results indicated that high acceptance strengthened the relation between ADHD and use of PCs.

The current study has limitations that need to be considered. First, the cross-sectional design did not allow us to examine the presence of causal relationship between ADHD, psychosocial factors, and ULBs. Future research could benefit from longitudinal designs to understand the temporal dynamics and causal effects of these relationships better. Moreover, ensuing studies should establish whether changes in specific psychosocial factors over time can affect the adoption of ULBs, further clarifying the possible causal link among variables, or the potential role of cultural and gender differences. Another limitation regards the sole use of self-report measures. Even though self-report questionnaires have several advantages (i.e., they are short and easy to administer, especially in large-scale studies), responses might not be immune to biases, such as social desirability and recall biases. Future research should therefore also include clinician-rated evaluations, more objective measures, or corroborative reports (e.g., reports from parents or teachers), which could enhance the accuracy of the data regarding ADHD symptoms and, thus, their interactions with ULBs. Finally, the Cronbach’s alpha for the total score of some scales (e.g., the PWB scales) could be considered somewhat low for psychological research. Future studies should further investigate the psychometric properties of the scales in this specific population or consider alternative instruments with higher reliability.

## 5. Conclusions

The current work aimed at investigating the presence of psychosocial moderators in the relationship between ADHD and ULBs.

The present findings carry important clinical implications, especially when developing interventions tackling psychological well-being, conducted by clinical psychologists, primarily addressed to adolescents with or without ADHD symptoms and secondarily to educators, other people working with them or families. Based on ADHD severity, such interventions, for example Well-Being Therapy [56], should aim at promoting a state of euthymia, which consists not only in the lack of mood disturbances but also in the balance of PWB dimensions reflecting, thus, the optimal level of psychological well-being to fulfill one’s own potential and self-realization [58]. Specifically, for adolescents with no or low levels of ADHD symptoms, clinical interventions should aim at improving psychological well-being dimensions and reducing risk factors such as allostatic overload, since our results indicated that it could help them in stemming ULBs. On the contrary, for adolescents with high ADHD symptomatology, clinical interventions should aim at balancing low perceived allostatic overload and the potential detrimental effect of too high levels of psychological well-being dimensions. Moreover, interventions should focus on the improvement of adolescents’ awareness regarding the negative consequences of ULBs, despite their perception of a general heightened well-being possibly deriving from the adoption of unhealthy lifestyle.

## Figures and Tables

**Table 1:** Descriptive statistics of the sample (N = 440).

Variables	N/Mean	%/(SD)
Biological sex (female)	240	54.5%
Age	14.21	(0.57)
Nationality (Italian vs. other)	399	90.7%
Mother’s level of education		
Elementary/Middle school	34	7.7%
High school	173	39.3%
Bachelor degree or higher	232	52.7%
Father’s level of education		
Elementary/Middle school	66	15%
High school	193	43.9%
Bachelor degree or higher	176	40%
Mother’s job status		
Retired	0	0%
Unemployed	8	1.8%
Employed	374	85%
Father’s job status		
Retired	6	1.4%
Unemployed	8	1.8%
Employed	408	92.7%
Family socio-economic status		
Poor	30	6.8%
Middle	332	75.5%
Rich	78	17.7%
High school attended		
Liceum A	110	25%
Liceum B	86	19.5%
Liceum C	76	17.3%
Technical institute A	56	12.7%
Liceum D	41	9.3%
Technical institute B	37	8.4%
Technical institute C	34	7.7%
Living with family members (vs. other)	363	82.4%
AUDIT-C—Alcohol Use Disorder Identification Test (Consumption)	1.50	(1.60)
HSI—Heaviness of Smoking Index	0.75	(1.35)
CAST—Cannabis Abuse Screening Test	5.37	(4.98)
Daily hours of sleep	6.90	(1.21)
Quality of sleep	2.72	(0.77)
Daily hours spent:		
Watching TV	7.67	(82.23)
Listening to the Radio	5.00	(67.33)
Using Smartphone	15.67	(105.57)
Using Tablet	7.45	(82.26)
Using PC	8.08	(82.21)
Playing Videogames	7.65	(82.24)
Daily hours spent using technological devices instead of sleeping	1.01	(1.32)
IAT—Internet Addiction Test (total score)	14.02	(3.94)
CSQL—Compromised Social Quality of Life	14.99	(4.67)
CIQL—Compromised Individual Quality of Life	10.27	(4.05)
CUI—Compensatory Usage of the Internet	5.96	(2.21)
CAWC—Compromised Academic/Working Careers	4.87	(2.15)
CTC—Compromised Time Control	6.01	(1.82)
EUI—Excitatory Usage of the Internet	4.10	(1.78)
ASRS—Adult ADHD Self-Report Scale (total score)	29.82	(10.46)
ASRS—Inattention	16.03	(6.18)
ASRS—Hyperactivity/Impulsivity	13.78	(5.54)
PSI-Y—PsychoSocial Index-Young		
Allostatic Overload (yes)	229	52%
PWB—Psychological Well-Being scales		
Self-Acceptance	7.93	(2.35)
Autonomy	8.42	(2.13)
Environmental Mastery	7.92	(2.19)
Personal Growth	8.93	(2.12)
Positive Relationship	8.74	(2.31)
Purpose in Life	7.99	(2.21)

Note. N = number; M = mean; % = percentage; SD = Standard Deviation.

## Data Availability

The data that support the findings of this study are available from the corresponding author (S.G.) upon reasonable request.

## Appendix A

**Table A1:** Correlations matrix among variables (N = 440).

	ASRS Total Score	IAT Compromised Time Control	PWBs Self-Acceptance	PWBs Environmental Mastery	PWBs Personal Growth	PWBs Positive Relations	PWBs Purpose in Life	Daily Hours Spent on Smartphone	Daily Hours Spent on PC
ASRS total score	-								
IAT Compromised Time Control	0.382 **	-							
PWBs Self-Acceptance	−0.306 **	−0.174 **	-						
PWBs Environmental mastery	−0.442 **	−0.367 **	0.443 **	-					
PWBs Personal growth	−0.102 *	−0.002	0.371 **	0.077	-				
PWBs Positive relations	−0.239 **	−0.124 **	0.506 **	0.341 **	0.254 **	-			
PWBs Purpose in life	−0.315 **	−0.207 **	0.411 **	0.466 **	0.371 **	0.291 **	-		
Daily hours spent on smartphone	0.263 **	0.228 **	−0.190 **	−0.241 **	−0.126 **	−0.103 *	−0.235 **	-	
Daily hours spent on PC	0.075	−0.007	0.005	−0.002	−0.042	0.016	−0.007	0.134 **	-
Daily hours spent using technological devices instead of sleeping	0.237 **	0.177 **	−0.147 **	−0.100 *	−0.189 **	−0.063	−0.251 **	0.340 **	0.147 **

Note. ASRS = Adult ADHD Self-Report Scale; IAT = Internet Addiction Test; PWBs = Psychological Well-Being scales. * *p* < 0.05; ** *p* < 0.01.
